# Comparative genomic study of non-typeable *Haemophilus influenzae* in children with pneumonia and healthy controls

**DOI:** 10.1016/j.isci.2024.111330

**Published:** 2024-11-06

**Authors:** Deming Zhang, Wenjian Wang, Chunli Song, Tingting Huang, Hongyu Chen, Zihao Liu, Yiwen Zhou, Heping Wang

**Affiliations:** 1 Shantou University Medical College, Shantou University, Shantou, Guangdong 515041, China; 2 Department of Clinical Laboratory, Shenzhen Traditional Chinese Medicine Hospital, Shenzhen, Guangdong 518033, China; 3 Department of Shenzhen Clinical College of Pediatrics, Shantou University Medical College, Shantou University, Shantou, Guangdong 518038, China; 4 Department of Clinical Laboratory, Shenzhen Hospital of Southern Medical University, Shenzhen, Guangdong 518101, China

**Keywords:** Disease, Genomics, Immunity

## Abstract

Non-typeable *Haemophilus influenzae* (NTHi) is a common pathogen causing respiratory infections, including pneumonia in children, and can also be found in the upper respiratory tracts of asymptomatic individuals. This study examines genomic variations between NTHi strains from healthy children and those from children with acute or chronic community-acquired pneumonia (CAP). Using bacterial genome-wide association studies (bGWAS), we compared these strains to identify key differences. Our analysis revealed that approximately 32% of genes exhibit variations between commensal and pathogenic states. Notably, we identified changes in *peptidoglycan biosynthesis* pathways and significant virulence factors associated with pneumonia. Furthermore, we observed a significant difference in β-lactam resistance due to PBP3 mutations between the healthy and pneumonia groups, confirmed by the ampicillin susceptibility test and characterized by the mutation pattern D350N, S357N, S385T, L389F. These findings contribute valuable insights into the genomic basis of NTHi pathogenicity and may inform more targeted clinical diagnostics and treatments.

## Highlights

• SNP patterns shift from healthy to disease stages, impacting pathogenicity
• bGWAS shows 32% of genes change from colonization to pneumonia
• Peptidoglycan pathway links to pneumonia pathogenicity
• PBP3 mutations for β-lactam resistance differ in health and pneumonia

## Graphical abstract

**Figure:**
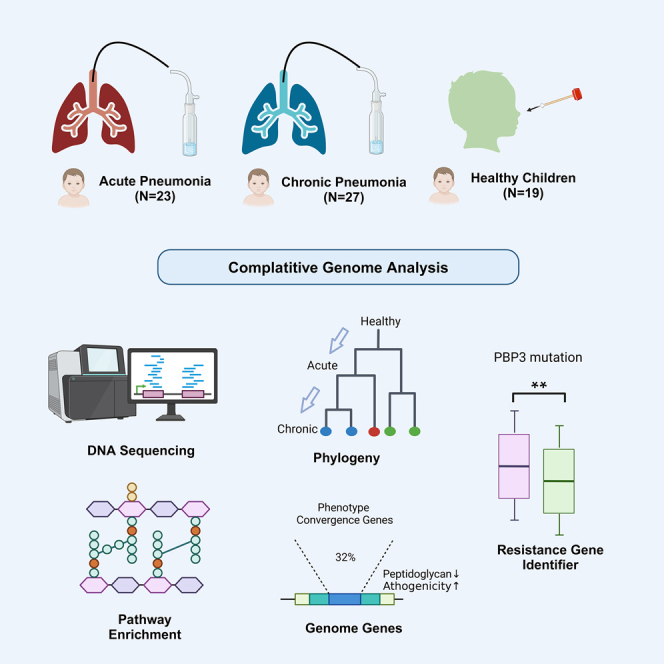

## Introduction

Childhood severe pneumonia is a serious respiratory disease with a high incidence and mortality rate among children in developing countries. To date, pneumonia remains one of the leading causes of death among all children who die before the age of five years as per etiologic investigations.^1^^,^^2^ The most common pathogens causing childhood pneumonia include *Streptococcus pneumoniae*, *Haemophilus influenzae* type b (Hib), *respiratory syncytial virus* (RSV), and *Mycoplasma pneumoniae*.^3^^,^^4^ In the past, invasive diseases caused by *Haemophilus influenzae* were often linked to strains with type b polysaccharide capsules. However, since the introduction of the Hib conjugate vaccine, there has been a significant decrease in the frequency of illnesses caused by this particular serotype.^5^^,^^6^ Worldwide, there has been a significant increase in invasive infections caused by non-vaccine-preventable *Haemophilus influenzae* strains. This increase is mainly attributed to NTHi strains.^7^^,^^8^

NTHi is an opportunistic pathogen of the upper respiratory tract in healthy children, which can infect the lower respiratory tract and induce chronic pulmonary diseases. Studies have shown that NTHi is the most commonly isolated bacterial pathogen in otitis media and sinusitis in children and one of the major drivers of acute exacerbation of chronic obstructive pulmonary disease (COPD) in adults.^9^^,^^10^ NTHi colonization/infection is also common in young children with cystic fibrosis.^11^

The molecular mechanisms behind the transition of NTHi from a commensal organism to a pathogen remain unclear. This shift may involve both internal changes within the organism and adaptations within the host. At the host level, when immune function is compromised, normally benign colonizers like NTHi can proliferate excessively and release significant amounts of inflammatory mediators, leading to infectious diseases. In this context, the bacteria are referred to as "opportunistic pathogens." Reduced immune function is thus a critical factor in the transition of NTHi from a commensal to a pathogenic state. For example, one study observed that even mild *influenzae* A virus infections can impair the host’s specific TH17 response to NTHi, increasing susceptibility to secondary bacterial infections.^12^

Further research has focused on bacterial strain variations. Some studies suggest that phase variation in NTHi is a mechanism underlying changes in its virulence. This includes homologous recombination, variations in simple sequence repeat lengths between allele variants, phase variation mediated by slip-strand mispairing, and transcriptional termination due to frameshift mutations.^13^^,^^14^^,^^15^ Some studies have shown that the phase variation of specific lipooligosaccharide (LOS) biosynthesis genes plays a critical role in the transition from colonizing the human nasopharynx to invading the middle ear cavity during the course of otitis media.^16^ Overall, there are relatively few genomic studies on the differences between NTHi from a commensal organism to a pathogen, partly because it is difficult to obtain pathogenic samples.

The present study aims to explore the genomic differences among NTHi strains isolated from the nasopharynx swabs of healthy children and bronchoalveolar lavage fluids (BALFs) of children with acute or chronic pneumonia. The objective is to uncover the genomic characteristics that enable NTHi to adapt to pulmonary infections and the underlying mechanisms through which NTHi causes acute and chronic pneumonia in children. This investigation is critical for enhancing the diagnosis, treatment, and prevention of NTHi-related lower respiratory tract diseases.

## Results

### Heterogeneity of NTHi genome across different clinical phenotypes

**Figure 1:**
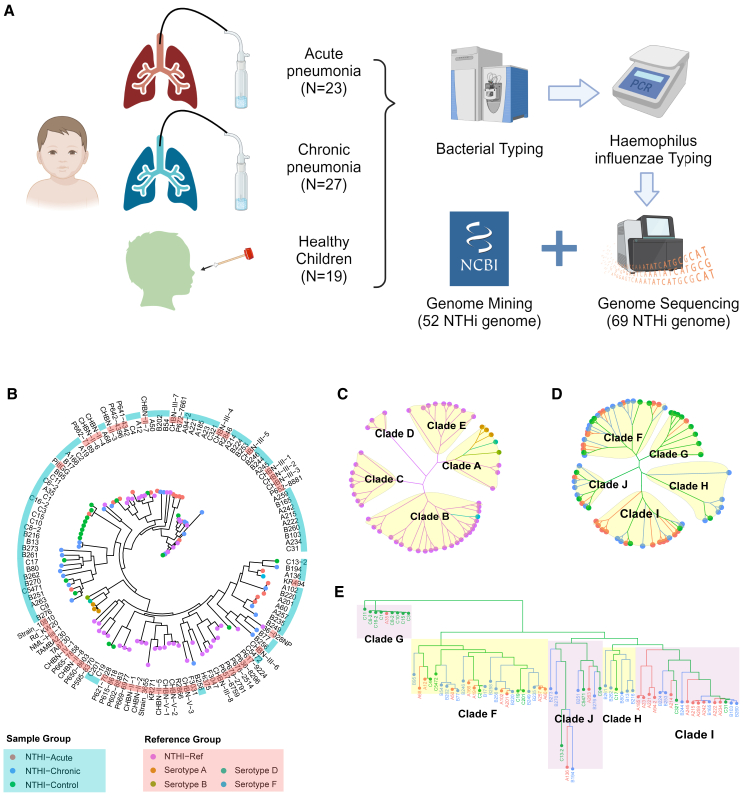
Sample collection workflow for phylogenetic tree analysis (A) Sample collection flowchart; 69 NTHi samples were acquired from this study, and 52 Haemophilus influenzae (Hi) reference sequences were sourced from the NCBI database. (B) A combination NJ tree of samples and HI references, present as Fan layout. The outer circle of the tree is colored as red and blue, representing the reference sequences and samples, respectively. The NJ tree tip label color scheme is as follows: red for acute cases, blue for chronic cases, green for healthy cases, orange for type a, brown for type b, teal for type d, and light blue for type f reference sequence. (C) NJ tree of 52 Hemophilus influenzae reference sequences, present as daylight layout. (D) NJ tree of 69 samples, present as daylight layout. (E) NJ tree of 69 samples, present as dendrogram layout, and root as Clade G. (C–E) using the same color scheme as (B).

The experimental design is outlined in Figure 1A. This study sequenced 69 samples to investigate the genomic characteristics of NTHi across different clinical phenotypes. The samples comprised 23 NTHi isolates from children with acute pneumonia, 27 from children with chronic pneumonia, and 19 from healthy children for comparison. Pneumonia samples (50 total) were obtained from BALF, whereas control samples (19 total) were collected from nasopharyngeal swabs. Next-generation sequencing yielded high-quality data with an average coverage depth of 1,161x (Table S1).

Phylogenetic tree analysis based on whole *Haemophilus influenzae* genomes from the NCBI database (*n* = 52, Table S2) and our own data (*n* = 69) reveals considerable genetic diversity, with the genomes divided into five main branches (clades A–E; Figures 1B and 1C). Within these branches, serotypes are intermixed: serotypes a, b, and d are found in clade A, whereas serotype f is located in clade E. This mixing may result from limited data for each serotype, as serotype a is closely clustered and distinct from other serotypes. Previous SNP-based studies identified six distinct NTHi clades, indicating vertical transmission of genetic information within lineages.^17^ Our results are consistent with these studies, showing genetic diversity and lineage evolution, though we categorized clades into five groups rather than six.

Secondly, although numerous samples in this study formed distinct clusters (as illustrated by the large connected groups of blue and red circles in Figure 1B), a discernible cross-cluster pattern with the database sequences remained evident. These findings suggest that the genomic variations between the samples in this study and those in the NCBI database are minimal, and no additional clades have been introduced to the NTHi phylogenetic tree.

Finally, the ancestral lineage of the 69 uncharacterized samples examined in this study was thoroughly analyzed with respect to their evolutionary relationships. The results revealed that both acute pneumonia samples (marked in red) and chronic pneumonia samples (marked in blue) can be traced back to the same lineage as the samples from healthy children (marked in green), as shown in Figure 1E. This suggests that NTHi pneumonia strains may have evolved from healthy strains or a common ancestor, acquiring specific traits or genetic changes that contributed to their pathogenicity. Although acute and chronic pneumonia strains, as well as strains from healthy children, do not cluster precisely in the evolutionary tree, the clustering pattern observed in Figure 1D indicates that the genomic variations associated with acute and chronic pneumonia may be complex, yet their patterns are still identifiable through evolutionary analysis.

### Certain genes in the genomes of acute and chronic NTHi pneumonia possess distinctive phenotypic effects

Based on the aforementioned analysis, bGWAS analysis was conducted using Hogwash software to identify genes significantly associated with phenotype variations, leveraging the relationship between genome and phenotype changes through convergent evolution.^18^

Pairwise comparisons indicated that the genes potentially associated with phenotype convergence were found within a range of 651–726 intervals across the three groups, encompassing 35.5%–39.6% of the reference genome, which consists of 1,834 genes (Table 1). This suggests that more than one-third of the genes may play a role in the phenotypic changes observed from health to acute pneumonia and chronic pneumonia.

**Table 1:** Phenotype convergence genes/regions

Groups	Annotated genes	Homology prediction genes	Intergenic region	Sum
Acute vs. Chronic	379	319	28	726
Acute vs. Control	339	293	29	661
Chronic vs. Control	333	279	39	657
Average	350	297	32	679

Note: Table 1 summarizes the genes and regions associated with phenotype convergence among three groups: Acute, Chronic, and Control. The table includes the number of annotated genes, genes predicted based on homology, and the intergenic regions for each comparison. The total count of identified genes and regions is provided in the "Sum" column for each comparison group. The average values across all groups indicate the overall trends in phenotype convergence.

When calculating the union of these groups, a total of 835 genes were identified as potentially linked to the dynamic evolution of NTHi phenotypes throughout the progression from health to acute pneumonia and eventually to chronic pneumonia (Table S3). Since NTHi is not yet a model organism for bacterial genomic research, many of its genes lack complete annotation and functional characterization. We classified all differentially expressed genes based on their annotation status and found that only about 40% of the phenotype convergence genes had clear functional annotations (Table S3). The remaining genes, often referred to as NTHi*_geneID*, are predicted by software but have unknown functions (Table S3). Our study focuses on the biological functions of genes with well-defined roles.

**Figure 2:**
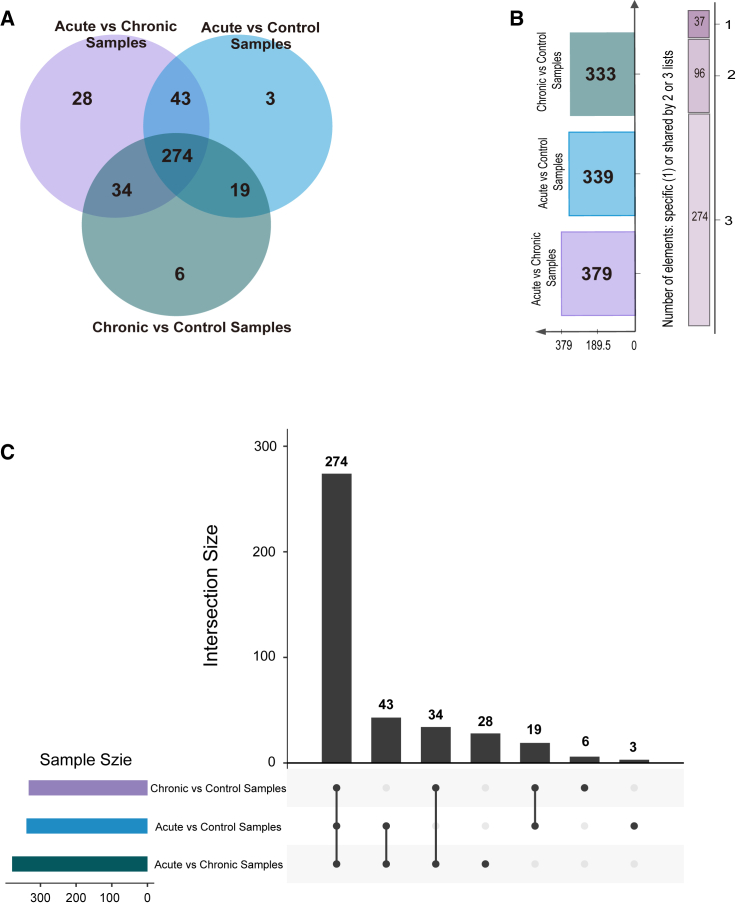
Relationship between phenotype convergence genes among different clinical groups (A) Venn diagram showing the phenotype convergence genes among acute, chronic, and healthy groups. (B) Bar chart and shared relationship plot of phenotype convergence genes among the three groups. (C) Upset view of phenotype convergence genes among the three groups. Table 1. Composition of annotated genes, homology-predicted genes, and intergenic regions in each clinical phenotype groups.

The Venn diagram analysis of intergroup genes identified 379 phenotype convergence genes between acute and chronic pneumonia, termed “chronic adaptive genes,” indicating adaptive genomic changes in NTHi from acute to chronic stages. There are 339 phenotype convergence genes between the acute pneumonia and healthy groups, referred to as “pathogenic transformation genes,” suggesting changes from colonization to pathogenicity. Additionally, 274 “Core genes” were identified as shared among all three groups, representing about 32.8% of the total phenotype convergence genes. These core genes reflect changes from health to acute or from acute to chronic stages, highlighting differences between NTHi colonization and infection (Figures 2A–2C).

### Multiple gene pathways involved in the pathogenicity and chronic adaptability of NTHi

**Figure 3:**
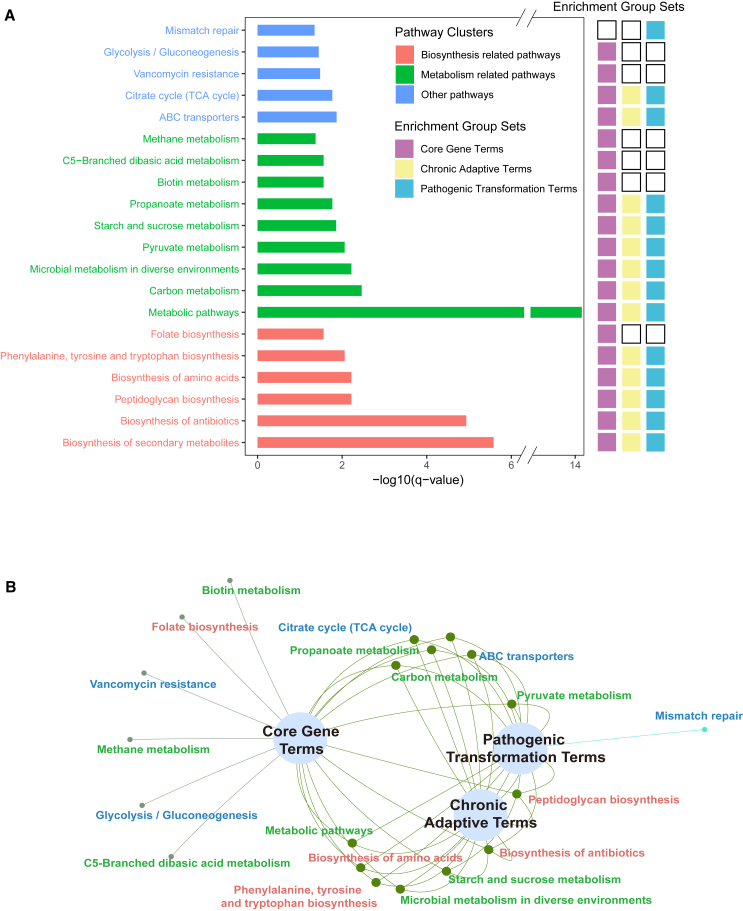
Enrichment analysis results of phenotype convergence genes in the three groups (379 chronic adaptive genes, 339 pathogenic transformation genes, 274 core genes) (A) Enrichment analysis results of KEGG displayed by bar plot. The results are classified into biosynthesis-related, metabolism-related, and others, represented by red, green, and blue, respectively. The right panel of Figure A shows whether each term is significantly enriched in different groups. Colored fillings indicate significant enrichment, with purple representing core genes, light yellow representing chronic adaptive genes, and light blue representing pathogenic transformation genes. (B) Network diagram of the enriched terms among the phenotype convergence genes in the three groups. Common pathways are linked, and individual pathways are represented as separate branches at the end. Color scheme is the same as in A.

Next, we conducted an enrichment analysis on three sets of phenotype convergence genes: chronic adaptive genes (379), pathogenic transformation genes (339), and core genes (274). Utilizing KOBAS 3.0, we performed the analysis through overrepresentation analysis (ORA), employing hypergeometric tests and Fisher’s exact tests to identify enriched pathways associated with the selected gene sets. As shown in Figures 3A and 3B, we identified 20 significantly enriched pathways, which were categorized into three groups based on their biological relevance. Although several pathways were enriched, most do not appear to be directly related to pneumonia phenotypes.

In the metabolism category, pathways such as *microbial metabolism under different environments* and *carbon metabolism* may have a more direct association with NTHi-induced acute and chronic pneumonia phenotypes, as they relate to bacterial survival and adaptation within the host environment, potentially influencing its pathogenicity. Within the biosynthesis category, *peptidoglycan biosynthesis* and *the biosynthesis of secondary metabolites* may also have direct connections to pneumonia phenotypes caused by NTHi. *Peptidoglycan synthesis* is crucial for bacterial cell structure, and any disruption in this process could affect NTHi’s biofilm formation, thereby impacting its survival and pathogenicity within the host. Additionally, the *biosynthesis of secondary metabolites* may play a role in modulating host immune responses and bacterial competition. In other categories, *glycolysis/gluconeogenesis* and *ABC transporters* may directly relate to pneumonia phenotypes induced by NTHi, as they are involved in bacterial energy metabolism and substance transport, which could influence NTHi’s survival and pathogenic capability within host cells.

Overall, the most significant pathway supported by previous evidence is the *peptidoglycan biosynthesis* pathway, which has the highest enrichment factor (Table S4). Prior studies have demonstrated that interference with NTHi’s peptidoglycan synthesis can enhance biofilm formation.^19^ Additionally, numerous studies suggest that biofilm formation by NTHi is a critical step in its pathogenic mechanism.^20^^,^^21^ We further analyzed the genes associated with this pathway, which are primarily composed of SNPs in *mraY*, *murA*, *dacB*, *murC*, *murD*, *murE*, *murF*, *murG*, and *mrcB*. It is worth noting that Mur enzymes (MurA-F) are key enzymes in peptidoglycan biosynthesis, playing a crucial role in bacterial survival and serving as important targets for antibiotic development.^22^ MurG, as a peripheral membrane protein interacting with phospholipids, may play a significant role in cell wall formation due to its oligomeric form.^23^

On the other hand, a comparison of the number of enriched pathways across different groups revealed that, despite the relatively small number of core genes (274), they were linked to more significantly enriched pathways than those associated with the pathogenic transformation (339 genes) and chronic adaptability (379 genes) groups. This suggests that core genes are frequently associated with critical pathway changes between phenotype changes.

### Analysis of virulence factors revealed seven specific virulence factors associated with NTHi pathogenicity

Although previous studies have identified various virulence factors in *Haemophilus influenzae*, a comprehensive genome-wide analysis has been lacking. In this study, we conducted a whole-genome scan of virulence factors using the VFDB database, performing comparative analyses among acute pneumonia, chronic pneumonia, and control groups. The VFDB, developed by the Chinese Academy of Medical Sciences, is a widely utilized resource for identifying virulence factor genes.^24^

**Figure 4:**
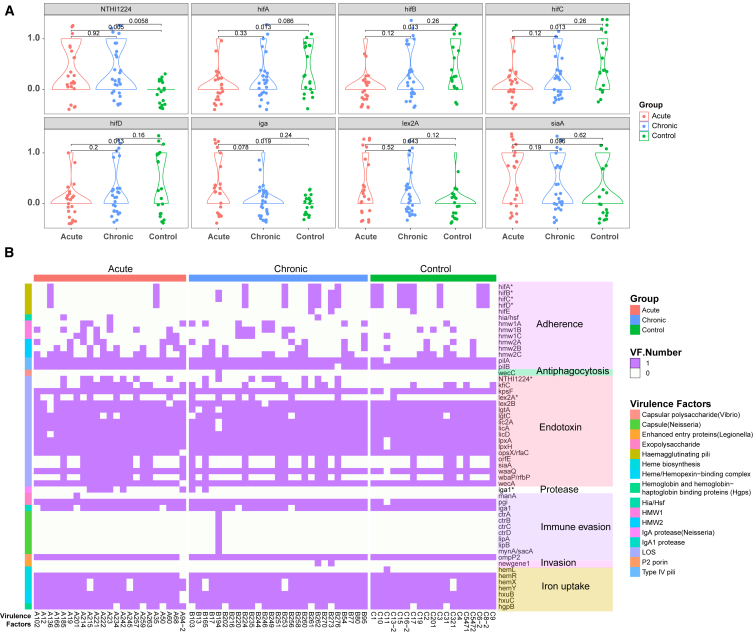
Virulence factor (VF) carriage among three clinical groups (A) Violin plots showing the difference in virulence factor carriage among three clinical groups. The *p* value between groups was calculated using the Wilcox test. Virulence factor carriage was categorized as present or absent, with 1 indicating presence and 0 indicating absence. The points in the violin plot were jittered for better visualization. Red represents the acute group, blue represents the chronic group, and green represents the healthy group. (B) Heatmap showing the virulence factor carriage among the three groups with VF classification annotations. The color fill in the heatmap represents the presence or absence of virulence factors, with purple indicating presence and white indicating absence. The primary level annotation of VF classification was directly labeled as text, and the second-level classification was represented by colored bars on the left side of the heatmap. The specific VF gene annotation was indicated in the row names of the heatmap. The colors of the three groups were the same as in Figure A.

The analysis revealed that seven out of eight classes of virulence factors were present in 69 NTHi samples, covering adherence, antiphagocytosis, endotoxin, protease, immune evasion, invasion, and iron uptake (Figure 4B). Toxin-related genes, such as *cdtA*, *cdtB*, and *cdtC*, were not detected, as they are specific to *Haemophilus ducreyi 35000HP*. Our samples also included virulence factor genes from NTHi serotype d, such as *oapA* and *ompP5*, suggesting inter-strain transmission of these factors. Notably, two samples from the chronic group contained an invasion-related gene not found in the NTHi reference genome (marked as *ngene*), likely derived from *Legionella*, which may enhance heterologous protein invasion.

In total, 53 virulence factor genes identified by this study were selected for differential analysis between clinical groups, including 16 virulence factors across 7 major classes. Seven genes (*hifA*, *hifB*, *hifC*, *hifD*, *NTHi1224*, *lex2A*, *iga*) that were identified exhibited significant differences between groups. The adhesion class genes (*hifA*, *hifB*, *hifC*, *hifD*) were found to differ between the acute and healthy groups, but not between acute and chronic groups, suggesting their role in the transition from colonization to infection rather than chronic adaptation. Additionally, the endotoxin genes *NTHi1224* and *lex2A*, encoding LOS, showed significant differences between the acute and control groups, with *NTHi1224* also differing in the chronic group. These results align partially with previous research and underscore the complexity of biological regulatory mechanisms and strain variability.^25^^,^^16^

The virulence factor of the protease class produced by the gene *igA1* also showed differences between the acute and control groups, and this virulence factor is believed to be associated with the occurrence of meningitis.^26^ Overall, the analysis conducted indicates that virulence factors may play a significant role in the variation of clinical phenotypes observed in NTHi. This finding may have important implications for the diagnosis, treatment, and prevention of NTHi infections, as understanding the mechanisms that underlie the pathogenicity of these bacteria is essential for developing effective interventions.

### Both the acute and chronic pneumonia groups commonly carry genes for resistance to cephalosporin antibiotics

**Figure 5:**
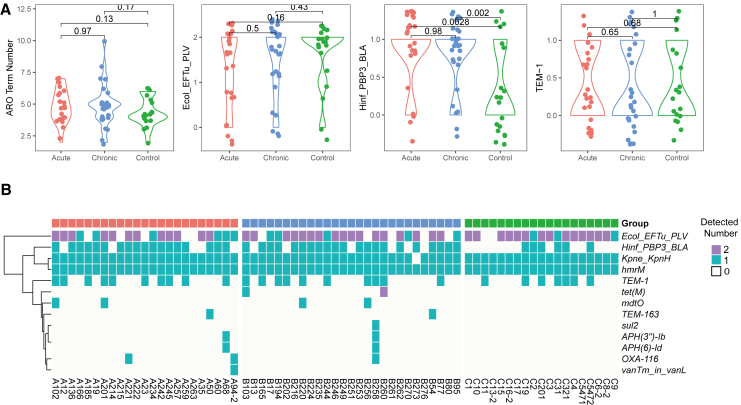
Genome-wide antibiotic resistance gene carriage in three clinical phenotypes (A) Differential analysis of ARO number, ARO gene family (CARD Short Name: Ecol_EFTu_PLV, Hinf_PBP3_BLA, TEM-1) among three clinical phenotypes. *p* values between groups were calculated using Wilcox test. (B) Heatmap of antibiotic resistance gene carriage. The color in the heatmap represents the number of AROs carried for a given drug resistance type. Purple represents two copies, cyan represents one copy, and white represents no copy. Red represents the acute group, blue represents the chronic group, and green represents the healthy control group.

To investigate the drug resistance characteristics of NTHi across different groups, we annotated all samples using the CARD database, which centers on ARO (Antibiotic Resistance Ontology) and includes terms related to antibiotic resistance genes, mechanisms, antibiotics, and targets.^27^ We first compared the identified ARO number among the three groups. Although the healthy group had lower ARO number (average 5.31) than the acute (5.86) and chronic pneumonia groups (5.92), there were no significant differences overall (Figure 5A).

A total of 13 Antimicrobial Resistance Ontology (ARO) items were identified in the NTHi genomes of this study (Table S5). The most frequently observed ARO item was Ecol_EFTu_PLV (ARO:3003369), associated with *Escherichia coli* EF-Tu mutants conferring resistance to Pulvomycin, detected in 57 of 69 samples. Despite its name suggesting an *E. coli* origin, this EF-Tu (elongation factor-Tu) mutation has been found in various bacteria, including *Haemophilus influenzae*. The frequency and number of this item in our samples were the highest, but no significant differences were detected among the identified groups.

*TEM-1* and *TEM-163* are β-lactamase genes capable of degrading various β-lactam antibiotics, such as penicillins and cephalosporins, thereby conferring antibiotic resistance to bacteria. *TEM-1* is a common prototype, whereas *TEM-163* is a variant with typically different resistance profiles. Our results indicate that most samples exhibit *TEM-1* resistance, with no significant differences observed between groups also.

Our study also detected resistance associated with altered PBP3. Amino acid substitutions in PBP3, caused by point mutations in the *ftsI* gene, can reduce binding of β-lactam antibiotics and lead to decreased susceptibility or resistance. We observed that this resistance was higher in both acute and chronic groups compared to the normal group, indicating a greater prevalence of PBP3 resistance mutations in disease-associated NTHi genomes. Previous research analyzing 91 *Haemophilus influenzae* isolates identified 14 different PBP3 amino acid substitutions. Common patterns included [D350N, M377I, A502V, N526K] (37.5%) in β-lactamase-positive rPBP3 strains and [D350N, A502T, N526K] (24.5%) in β-lactamase-negative rPBP3 strains.^28^

Next, we compared the prevalence of identified antibiotic resistance items with those documented in the NCBI database for *Haemophilus influenzae* and other 412 important pathogens, defined by RGI, as detailed in Table 2 and Table S6. The majority of resistance items aligned well between our study and the database. However, significant discrepancies were noted for two items: the KpnH efflux pump, present at 98.55% in our study versus 19.59% in the database, and the hmrM efflux pump, detected at 100% in our study and only 1% in the database. These differences suggest that NTHI may have distinct resistance mechanisms compared to *Haemophilus influenzae*.

**Table 2:** Prevalence comparison antibiotic resistance item

CARD Short name	Prevalence in this study	Prevalence of *Haemophilus influenzae* in NCBI chromosome	Representative high prevalence strain (with prevalence of NCBI chromosome)
APH(3″)-Ib	2.90%	1.03%	*Klebsiella huaxiensis* (100%)
APH(6)-Id	2.90%	1.03%	*Klebsiella huaxiensis* (100%)
Ecol_EFTu_PLV	100.00%	100%	*Klebsiella pneumoniae* (97%)
Hinf_PBP3_BLA	65.22%	41.24%	*Klebsiella pneumoniae* (99%)
Kpne_KpnH	98.55%	19.59%	*Klebsiella pneumoniae* (99%)
OXA-116	4.35%	Not Report	Not Report
TEM-1	39.13%	10.31%	*Klebsiella huaxiensis* (100%)
TEM-163	2.90%	Not Report	Not Report
hmrM	100.00%	1.03%	Not Report
mdtO	5.80%	Not Report	*Shigella sonnei* (98%)
sul2	1.45%	Not Report	*Klebsiella huaxiensis* (100%)
tet(M)	4.35%	Not Report	*Streptococcus pseudopneumoniae* (100%)
vanTmL	1.45%	Not Report	Not Report

Note: Table 2 displays the prevalence of various antibiotic resistance genes identified in this study versus their prevalence in *Haemophilus influenzae* as recorded in the NCBI database (Detail see in Table S6). The table also includes information on representative strains with high prevalence of the respective genes, where available. Prevalence is expressed as a percentage.

Additionally, certain resistance items, such as APH(3″)-Ib, APH(6)-Id, sul2, and vanTmL, were found at low frequencies in both NTHI and *Haemophilus influenzae*. These items likely originated from other bacteria through horizontal gene transfer or plasmid exchange. For example, *Klebsiella huaxiensis*, a less virulent pneumonia-causing bacterium, harbors the sul2 item at a 100% rate, compared to only 1.45% in our study. This highlights the potential for resistance gene exchange among bacteria to significantly influence the development and spread of antibiotic resistance.

### A PBP3 mutation pattern is linked to children pneumonia caused by NTHI

**Figure 6:**
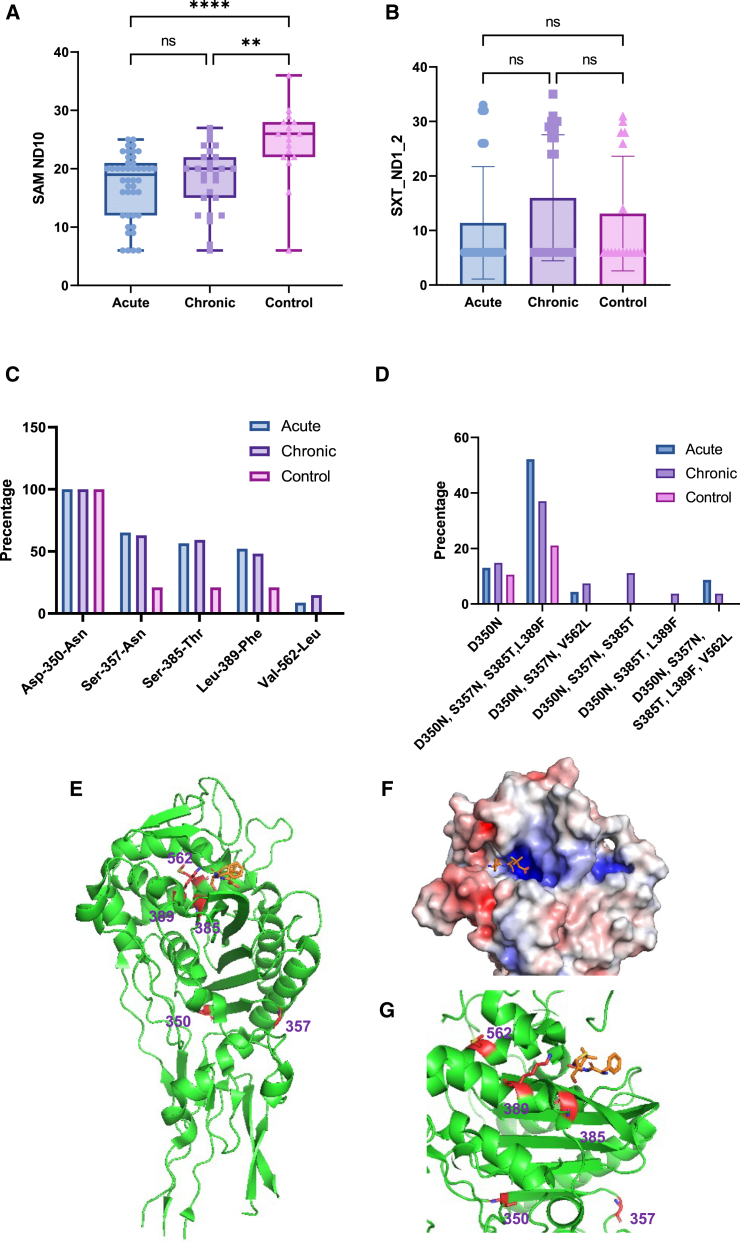
Analysis of NTHI resistance and PBP3 mutation sites (A) Comparative analysis of SAM_ND10 levels among acute, chronic pneumonia, and control groups. The graph displays the average values for each group, with error bars indicating the standard deviation. Significance is denoted by asterisks, where ns indicates non-significance, ∗∗∗∗ indicates highly significant (*p* < 0.0001), ∗∗ indicates significance (*p* < 0.01), and ∗ indicates moderately significant (*p* < 0.05). (B) Comparative analysis of SXT_ND1_2 levels among acute, chronic pneumonia, and control groups. The graph displays the average values for each group, with error bars indicating the standard deviation. Non-significance is indicated by ns. (C) Illustrates the mutation sites and types of amino acid mutations detected in PBP3 protein in this study. The y axis represents the frequency of each mutation site in the samples. (D) Shows the combination patterns of mutation sites detected in PBP3 protein. The y axis represents the frequency of each combination pattern in the samples. (E) Structural diagram of PBP3 protein with ampicillin (PDB:6kgw). The PBP3 protein is depicted in green, ampicillin in orange, and the mutation sites detected in this study are marked in dark red. (F) Represents the electrostatic potential distribution map of the binding site between PBP3 protein and ampicillin. Blue areas indicate positive charges, and red areas indicate negative charges. (G) Shows different perspectives of the binding site between PBP3 protein and ampicillin.

We found that the PBP3 mutation rate was higher in both acute and chronic pneumonia patients compared to healthy individuals. To investigate the impact of these mutations on drug resistance, we tested the susceptibility of NTHi strains to various antibiotics. The results revealed significant differences in the MIC (minimum inhibitory concentration) for compound antibiotics (ampicillin/sulbactam) among the pneumonia groups, with acute and chronic patients showing greater resistance than the controls. However, there was no significant difference in resistance to the broad-spectrum antibiotic STX (trimethoprim/sulfamethoxazole; Figures 6A and 6B).

This outcome is expected because ampicillin is a broad-spectrum beta-lactam antibiotic, and sulbactam inhibits beta-lactamases; PBP3 mutations could impair their efficacy. Conversely, co-trimoxazole hinders bacterial replication by disrupting folate metabolism, a process less affected by PBP3 mutations.

Comparing our findings with previous research, we noted similarities in PBP3 mutation sites, although patterns specific to NTHI PBP3 have not been extensively studied. We identified five mutation sites and six mutation patterns in PBP3 (Figures 6C and 6D; Table S7). All sites were previously reported,^29^^,^^30^^,^^31^^,^^32^ but the high-frequency patterns (D350N, S357N, S385T, L389F) in our study have not been previously discovered (Table S7). This combination was most prevalent in acute patients (52.17%), followed by chronic (37.04%), and healthy individuals (21.05%).

We also explored PBP3’s structure in complex with ampicillin (Figure 6E) and revealed that the mutation sites 389 and 385 are crucial for the antibiotic’s binding pocket (Figure 6F). This suggests that mutations at these sites may have a key impact on reducing the antibiotic’s binding affinity, thus diminishing its effect on bacterial cell wall synthesis. Additionally, D350N and S357N might be critical for PBP3’s interaction with other proteins (Figure 6G), potentially influencing drug resistance.

## Discussion

In this study, we examine single nucleotide polymorphisms (SNPs) in the NTHi genome across various clinical groups to identify genomic differences potentially associated with clinical phenotypes. Although single-omics approaches have their limitations, DNA remains a critical determinant of gene expression and function. Consequently, analyzing SNP distribution and variation is essential for identifying genetic factors linked to clinical outcomes. Through phylogenetic analysis of bacterial genomes, we confirmed the pattern of SNP variations occurring from healthy to acute and then to chronic stages. This result supports the hypothesis that genomic variations in bacteria contribute to alterations in pathogenicity. Utilizing bGWAS analysis, we assessed the proportion of genetic variations during the progression from colonization to acute and chronic pneumonia, revealing that approximately 32% of genes exhibited changes during this phenotypic transition.

In phenotype convergence gene enrichment analysis, we identified a significant association between the peptidoglycan biosynthesis pathway and the pathogenic phenotype of pneumonia. Previous *in vitro* study has demonstrated that disrupting peptidoglycan synthesis in NTHi can lead to increased bacterial lysis and elevated extracellular DNA levels, which in turn promotes biofilm formation.^19^ The formation of biofilms enhances NTHi’s resistance to antibiotics and the host immune system, playing a critical role in the bacterium’s colonization, persistence, and chronic recurrent infections.^21^^,^^33^ Notably, biofilms of NTHi have been observed in the lower respiratory tracts of children with otitis media and patients with chronic obstructive pulmonary disease (COPD),^21^ the aforementioned study all suggesting that biofilms may play a central role in the pathogenesis of these conditions.

Interestingly, the study also found that similar biofilm-promoting effects could be achieved with sub-inhibitory concentrations of β-lactam antibiotics, whereas other classes of antibiotics did not exhibit this effect.^19^ This implies that β-lactam antibiotics (particularly against β-lactam-resistant NTHi strains) might facilitate biofilm formation, thereby enhancing bacterial resistance to antibiotics. This finding is consistent with our RGI analysis results, which revealed a significantly higher incidence of β-lactam antibiotic-resistant PBP3 mutations in patients with acute and chronic pneumonia compared to healthy controls. This suggests a potential association between PBP3 mutations and pneumonia, likely mediated through biofilm formation. Furthermore, recent research has demonstrated that NTHi strains with altered PBP3, characterized by specific amino acid substitutions such as N526K, exhibit an increased capacity to invade respiratory epithelial cells, which could be a critical factor in the pathogenesis of pneumonia.^34^ Notably, another study has also shown that certain amino acid substitutions in PBP3 are associated with an increased invasion of bronchial epithelial cells by *Haemophilus influenzae*.^35^ These findings collectively underscore a potential association between PBP3 mutations and pneumonia, possibly mediated through enhanced biofilm formation and subsequent antibiotic resistance.

Additionally, PBP3 is a crucial enzyme in peptidoglycan synthesis (as detailed in the literature on Peptidoglycan Biosynthesis and Regulatory Mechanisms).^36^ Based on the combined insights from our findings, it is reasonable to speculate that PBP3 mutations are not only associated with β-lactam antibiotic resistance but also with the formation of NTHi biofilms. Specifically, PBP3 plays a pivotal role in peptidoglycan synthesis, and mutations in this enzyme may alter peptidoglycan biosynthesis, thereby affecting the integrity and structure of the bacterial cell wall and promoting biofilm formation. This, in turn, increases NTHi’s resistance to antibiotics, complicating treatment efforts.

Furthermore, disruptions in peptidoglycan synthesis and PBP3 mutations may enhance bacterial survival and colonization within biofilms, explaining the higher prevalence of these mutations in patients with acute and chronic pneumonia. Biofilms not only impede antibiotic penetration but also shield bacteria from host immune responses, thereby diminishing the effectiveness of antibiotic treatment.^37^ Therefore, PBP3 mutations and their impact on biofilm formation are likely key factors contributing to increased NTHi resistance. Further research into the specific mechanisms linking PBP3 mutations to biofilm formation will be crucial for understanding NTHi’s pathogenicity in diseases such as pneumonia and for developing more effective treatment strategies.

Comparing our findings with existing databases and previous studies, we found that most of the identified resistance genes in this study match the rates seen in *Haemophilus influenzae*. However, there were notable differences in the occurrence of two antibiotic efflux pump items, KpnH and hmrM. A study in Guangzhou, China, also reported a 100% detection rate for hmrM^38^ in their NTHi samples, suggesting that a potentially emerging branch of the NTHI strain may be circulating widely in southern China. Although all PBP3 mutations identified in this study have been previously documented, the specific combinations of these mutations in NTHI are less commonly reported. In our research, a particular combination of PBP3 mutations (D350N, S357N, S385T, L389F) was not only prevalent but also varied significantly across different clinical presentations of pediatric pneumonia. This insight could be crucial for understanding NTHI’s resistance mechanisms and informing clinical treatment approaches.

Of course, we must acknowledge that there are certain limitations to this study, such as the incomplete recording of antibiotic use during the collection of clinical samples, as well as the time lag between sampling and drug administration. If these factors can be better controlled, we may be able to further investigate the mechanisms of antibiotic resistance and its formation in more depth. Additionally, the study lacks animal experiments using pneumonia models to validate the genetic findings at a phenotype level. Nevertheless, overall, this study represents a comparative genomic analysis of NTHi across multiple clinical phenotypes, with potential guiding significance for NTHi genomics and clinically related research.

### Limitations of the sstudy

The comprehensiveness of our antibiotic resistance analysis may be influenced by the incomplete recording of antibiotic usage at the time of clinical sample collection. Furthermore, the temporal gap between sampling and drug administration adds a layer of complexity to the exposure assessment. Enhanced by the absence of pneumonia models in animal studies, the phenotypic confirmation of genetic data is similarly limited.

## Resource availability

### Lead contact

Further information should be directed to and will be fulfilled by the lead contact, Dr. Heping Wang (szetgmy@163.com).

### Materials availability

This study did not generate new unique reagents.

### Data and code availability

• All other data reported in this paper will be shared by the lead contact upon request.
• The raw sequencing FASTQ format data have been deposited in the CNGB Sequence Archive (CNSA) of China National GeneBank DataBase (CNGBdb) and have been made publicly available since October 4, 2024. Accession numbers are listed in the key resources table.
• This paper does not report the original code. Any additional information required to reanalyze the data reported in this paper is available from the lead contact upon request.

## Acknowledgments

We want to thank Prof. Zuguo Zhao for guidance in our research, which has significantly shaped our understanding and approach. This work was supported by Guangdong High-level Hospital Construction Fund [ynkt2021-zz10], Shenzhen Fund for Guangdong Provincial High-level Clinical Key Specialties [SZGSP012], and Shenzhen Key Medical Discipline Construction Fund [SZXK032]. Shenzhen Science and Technology Program [JCYJ20220530154210022].

## Author contributions

H.W. and W.W. conceived the project and designed the experiments. Z.L. and D.Z. collected mRNA samples and conducted PCR and mass spectrometry analysis. D.Z., C.S., and T.H. performed mRNA sequencing, GWAS analysis, and functional enrichment analysis. D.Z. drafted the manuscript, incorporating feedback from all authors. Y.Z. supervised the project and revised the manuscript. All authors approved the final version.

## Declaration of interests

The authors declare that they have no competing interests.

## STAR★Methods

### Key resources table

**Table:** 

REAGENT or RESOURCE	SOURCE	IDENTIFIER
**Bacterial and virus strains**		

*Haemophilus influenzae* strain AMC 36-A-1	ATCC	ATCC 10211
Clinical Isolated *Haemophilus influenzae* strains	Shenzhen Children’s Hospital	N/A

**Biological samples**		

Nasopharyngeal swabs	Shenzhen Children’s Hospital	N/A
Bronchoalveolar lavage fluids	Shenzhen Children’s Hospital	N/A

**Critical commercial assays**		

MGIEasy Fast Enzymatic DNA Library Prep Kit	MGI	Cat#940-000027-00
Conventional cyclization reagent kits	MGI	Cat#1000020570
MGISEQ-2000RS high-throughput sequencing reagent kit	MGI	Cat#1000012555

**Deposited data**		

HTHi Sequencing Date for this study	CNGB Sequence Archive (CNSA)	CNP0005331

**Software and algorithms**		

fastp	Shifu Chen et al., 2023	https://github.com/OpenGene/fastp
bwa-mem	Li et al., 2009	http://bio-bwa.sourceforge.net/bwa.shtml
Genome Analysis Toolkit (GATK)	Broadinstitute	https://github.com/broadinstitute/gatk/
MUMmer4	Marçais et al. 2018^39^	https://mummer4.github.io/
Fasttree	Price et al. 2010^40^	http://www.microbesonline.org/fasttree/
hogwash	Saund and Snitkin, 2020^18^	https://github.com/katiesaund/hogwash
SnpEff	Cingolani et al. 2012^41^	https://pcingola.github.io/SnpEff/
SnpSift	Cingolani et al. 2012^42^	https://pcingola.github.io/SnpEff/#snpsift
EVenn	Mei Yang et al., 2024	http://www.ehbio.com/test/venn/#/
KOBAS-i	Bu et al. 2021^43^	http://bioinfo.org/kobas
VFDB VFanalyzer	Liu et al. 2022^24^	http://www.mgc.ac.cn/cgi-bin/VFs/v5/main.cgi
RGI	Alcock et al. 2023^27^	https://card.mcmaster.ca/analyze/rgi
R 4.0.4	R 4.0.4	https://www.R-project.org/

### Experimental model and study participant details

#### Ethical approval and consent

This study was approved by the Ethical Committee of Shenzhen Children’s Hospital with registration number 2016013. Written informed consent for the storage and use of the BAL or NP samples for further studies was obtained from the parents or caregivers before enrollment.

#### Study participants and sample collection

The clinical samples used in this study were obtained from the sample library of the Clinical Microbiology Laboratory at Shenzhen Children’s Hospital, with a collection period spanning from February 2019 to October 2021. The samples comprised *Haemophilus influenzae* strains derived from two distinct groups: the nasopharynx of healthy children and the bronchoalveolar lavage fluids of hospitalized children. The healthy children’s samples were collected via nasopharyngeal swabs during routine health examinations, totaling 19 samples for comparison. In contrast, the hospitalized children, who were diagnosed with either acute or chronic respiratory infections, provided bronchoalveolar lavage fluids. This group was further divided into two categories based on the duration of their symptoms: 23 samples from children with acute pneumonia, characterized by symptoms developing within one month, and 27 samples from those with chronic pneumonia, defined as symptoms persisting for over three months. All children included in the study presented clinical symptoms of pneumonia, such as coughing, chest tightness, wheezing, and fever, along with distinctive pulmonary imaging features. Gender and age details are provided in Table S1. While there are minor age variations among phenotype groups, previous research suggests that both age and gender typically influence microbial diversity. However, no studies have reported an effect of these factors on the genomic DNA of different bacterial strains.

### Method details

#### Sample collection, strain identification, and identification of NTHi types

The lavage fluid was subjected to routine bacterial culture, with the specimens inoculated onto *Haemophilus influenzae* selective plates and Columbia blood agar plates (both purchased from bioMérieux, France) and incubated at 35°C under a 5% CO_2_ atmosphere for approximately 24 h. After incubation, small, dewdrop-shaped, colorless, and transparent colonies were selected as suspected colonies. Gram staining was performed, with the results showing short, small, and gram-negative rods. Bacterial colonies were picked and plated onto VITEK MS-DS target plates, followed by matrix solution addition for lysis, air-drying, and on-machine analysis of the results. All isolated clinical strains were subjected to MALD-TOF (matrix-assisted laser desorption/ionization time-of-flight mass spectrometry) for mass spectrum acquisition and analyzed using the Merk VITEK MS database for microbiological identification, confirming the identity of the strains as *Haemophilus influenzae*. Total bacterial DNA was extracted from the *Haemophilus influenzae* and PCR detection of the *Haemophilus influenzae* capsule gene (bexA) was performed. The strain *Haemophilus influenzae* of type b, ATCC10211, was used as a positive control, and gel electrophoresis was employed to observe the PCR products, facilitating the classification of *Haemophilus influenzae*. Strains that did not show the target band were determined to be NTHi and included in this study.

#### Extraction, library preparation, and sequencing of the genome

The extraction of the sample genome was completed using the MGIEasy Fast Enzymatic DNA Library Prep Kit (catalog number: 940-000027-00), which is compatible with a variety of single-bacteria and meta-sample library preparations and is suitable for microbial WGS, meta-species identification, abundance determination, and assembly. Conventional cyclization reagent kits (MGI, catalog number: 1000020570) were used to cyclize the obtained PCR library to obtain single-stranded circular DNA. Conventional make DNB was performed using the make DNB reagent components in the MGI sequencing reagent kit. After the library was prepared, the MGISEQ-2000RS high-throughput sequencing reagent kit (FCL PE150) was used for genome sequencing (catalog number: 1000012555). The MGISEQ-2000RS high-throughput sequencing reagent kit uses combinatorial probe-anchor synthesis (cPAS) technology to aggregate DNA molecules and fluorescent probes on DNA nanospheres (DNBs), and high-resolution imaging systems are used to collect optical signals that are digitized to obtain high-quality and accurate sample sequence information.

Extract the genomic DNA and randomly break it into fragments, perform electrophoresis to recover DNA fragments of the required length, add adapters for cluster preparation, and finally perform sequencing. After the DNA samples are received, they are checked, and qualified samples are used to construct libraries: first, large DNA fragments are randomly fragmented into 500-800bp fragments using ultrasound methods such as Covaris or Bioruptor. The sticky ends generated by the fragmentation are repaired into blunt ends using T4 DNA Polymerase, Klenow DNA Polymerase, and T4PNK. Then, the 3′ end is extended with an “A” base so that the DNA fragment can be connected to a special adapter with a 3′ end carrying a “T” base. The target fragments are selected using electrophoresis to connect the products, and PCR technology is used to amplify the DNA fragments with adapters at both ends. Finally, qualified libraries are used for cluster preparation and sequencing.

#### Organizing and selecting the NTHi reference genome for the database

To determine the genomic status of NTHi in the database and select representative strains as the reference sequence for this analysis, we compiled all NTHi genomes in the existing NCBI nucleotide database and The Reference Sequence (Refseq) database. A total of 107 sequences between 1.6M and 2.4M nucleotide lengths were collected and compiled for this study. As the genome of NTHi was first sequenced and evaluated as 1.83M,^44^ theoretically, the sequences in our selected length range should represent complete or nearly complete NTHi sequences. We found that nine of the 107 sequences were deleted or removed from Refseq due to quality issues. Notably, the removed sequences include strains frequently mentioned in previous studies, such as NC_009566.1 (PittEE) and NC_009567.1 (PittGG) (see Table S1). Among the remaining sequences, 86 were identified as NZ - not finished WGS sequences, although many of them had titles suggesting complete genomes. One sequence was only available in GeneBank. Finally, a total of 11 sequences were identified as NC-level genomes.

We further manually curated and annotated the typing of the 107 sequences and found that most strains listed in NCBI did not adopt a unified typing standard. Previous studies have also shown that there is no unified standard for typing this bacterial sequence due to its diversity.^45^ Nonetheless, most previous studies have identified the typing of NTHi through serotype, MLST typing, or capsule expression. Therefore, we finally selected 52 genomes with serotype annotations for evolutionary comparative analysis. For genome assembly with a reference genome, we used five NTHi strains at the NC (complete genome) level as reference sequences. For SNP analysis and gene annotation, to maintain consistency with previous studies, we used the 86-028NP strain as the reference sequence for analysis.

#### Filtering, alignment, SNP analysis, and construction of the evolutionary tree for the sequences

The downstream data from the aforementioned sequencing was first filtered using fastp software (version 0.20.1)^46^ to remove adapters and low-quality sequences. Specifically, since the subsequent sequences needed to be used for assembly, we used the parameter --trim_poly_x --poly_x_min_len 10 to filter the poly sequences to reduce their impact on subsequent assembly analysis. After data filtering, we used bwa-men (Version: 0.7.17-r1188)^47^ to perform sequence alignment and SNP and Indel analysis based on the selected 86-028NP reference genome. The mutect2 module in the Genome Analysis Toolkit (GATK) v4.1.1.0^48^ was used for SNP and Indel analysis based on the reference genome. Even though GATK has an SNP and Indel variant calling pipeline called GATK-for-Microbes for bacterial reference genomes, we conducted tests and discovered that Mutect2 produced more reliable results. Therefore, we selected Mutect2 for our study.

After obtaining the SNPs for each sample through GATK, we used an internal script to construct a consensus sequence for each sample based on the reference genome. On the other hand, for the selected 52 complete reference genome sequences of NTHi, MUMmer4^39^ was used to call SNPs based on the reference sequence and construct the consensus sequence. Finally, we combined the consensus sequences of the samples and the reference sequences and used the software fasttree (version 2.1.10)^40^ to construct the NJ evolutionary tree. After obtaining the tree file of the evolutionary tree through fastree, we used the ggtree^49^ package in R software to display and beautify the evolutionary tree and used the ggtreeExtra,^50^ ggstar,^51^ and ggnewscale^52^ packages to add annotations and layers to the evolutionary tree.

#### NTHi whole-genome association analysis and acquisition of phenotype-convergent genes

The whole-genome association analysis of NTHi was performed using the R package hogwash.^18^ Based on a comparison between the Phyc and Synchronous algorithms that come with the software, the phenotype-convergent genes obtained from the Synchronous algorithm developed by the software were selected for subsequent analysis. We used the post-ancestral reconstruction grouping algorithm included in the software to perform gene- and differential-based grouping and analysis of SNPs. The SNP gene annotation was completed using SnpEff version 5.0e^41^ based on the reference genome NC_007146.2, and SnpSift^42^ was used to extract the relationship between SNPs and genes after gene annotation was completed. The homology-predicted functionally unknown genes in the reference genome, i.e., in 86-028NP, were labeled based on the NTHi-GeneID pattern.

In particular, some SNPs are annotated as existing in intergenic regions, so intergenic regions are also treated as independent units for testing whole-genome association. Therefore, the phenotype-convergent genes identified in this study are divided into three types for display: annotatable, homology-predicted, and intergenic regions. In this study, the phenotype refers to three clinical differentiations: acute, chronic, and healthy. After obtaining the phenotype-convergent genes, the online software EVenn^53^ (http://www.ehbio.com/test/venn/#/) was used to display the differences between groups. We used two modules, Interactive Venn Diagram and Upset Plot, to show the differences in gene expression between groups from different perspectives. After uploading the raw data to EVenn to obtain the original SVG-format image, Adobe Illustrator was used to integrate the images.

#### Enrichment analysis of phenotype-convergent genes

The enrichment analysis of phenotype-convergent genes was performed using the Gene-list Enrichment module in the online analysis software KOBAS-i (http://kobas.cbi.pku.edu.cn/),^43^ with *Haemophilus influenzae 86-028NP (nontypeable)* selected as the species. Although KOBAS-i supports four pathway databases, KEGG Pathway (K), Reactome , BioCyc (B), and PANTHER §, as well as GO enrichment analysis, only the KEGG Pathway database is supported for the species of NTHi. On the other hand, we attempted to use gene IDs similar to NTHi-GeneID for gene annotation in KOBAS, but none of the NTHi genes were collected in the KEGG database. Therefore, we excluded genes starting with NTHi and selected annotated genes for pathway enrichment analysis. After obtaining the enrichment results, the Gene List Enrichment Visualization module was used to obtain the raw data for pathway associations and Rich Factor for each pathway. Furthermore, these data were displayed using the pheatmap package^54^ in R and the Venn Network module in EVenn.^53^

#### Genome assembly, polishing, and annotation

Genome assembly was based on filtered data from downstream sequencing, and SPAdes (v3.13.0)^55^ was used for genome reassembly. Trust-contig was set for the aforementioned five high-quality NTHi genomes starting with NC, including NC_007146.2 (86-028NP), NC_014920.1 (F3031), NC_014922.1 (F3047), NC_017451.1 (R2866), and NC_017452.1 (R2846). The kmer lengths used for assembly were the software default of 33, 55, and 77 bp, and the final result was based on the optimal N50 length. In total, around 40 scaffolds with an N50 length of approximately 11K were obtained for each sample. To further obtain a draft of the NTHi whole genome for each sample, Ragout (Reference-Assisted Genome Ordering Utility) (V2.3)^56^ was used for scaffold splicing. The basic principle was to construct an A-Bruijn graph by using Cactus to align genomes and obtain colinear blocks, which were then reconstructed into a chromosome-level genome. The reference in the recipe_file was set to the single reference genome NC_007146.2 (86-028NP), and the scaffolds obtained from SPAdes were used for input. Finally, complete draft genome sequences with some N regions were obtained for all samples, and the genome sizes ranged from 1.8M to 2.2M. After completing the draft genome assembly, Prokka (rapid prokaryotic genome annotation, v1.14.6)^57^ was used for annotation of the whole genome, and the kingdom level selected was Bacteria.

#### Toxicity and drug resistance analysis

Analysis of virulence factors was based on the VFDB (Virulence Factor Database)^24^ database and was performed using the online analysis software VFanalyzer (http://www.mgc.ac.cn/cgi-bin/VFs/v5/main.cgi). The “Select genus of the genome” was set to *Haemophilus*, “Strain name” was set to the sample name, and “upload type” was set to “Pre-annotated DRAFT genome in GenBank format”. The input sequence was the genome draft obtained from the Ragout assembly, and the representative genome was set as NC_007146.2 (86-028NP). After online analysis was performed for all samples, the analysis results were downloaded and summarized locally. The summarized results were displayed using the R package “pheatmap”^54^ for heatmap visualization, “ggboxplot” function of “ggplot2”^58^ for boxplot visualization between groups, and “ggpubr”^59^ for calculating the significance of differences between groups. The significance of differences between groups was determined using the Wilcox test.

Analysis of drug resistance genes was based on the CARD database (https://card.mcmaster.ca/)^27^ and was performed using the main module of the Resistance Gene Identifier (RGI) software, with the search mode set to DIAMOND. After obtaining the ARO entries of resistance genes for each sample, custom scripts were used to count ARO data and drug class features for each sample. The display of statistical results was also based on the R package “pheatmap”^54^ and the “ggpubr”^59^ package.

#### Antibiotic susceptibility testing

Select well-cultivated pure cultures and adjust the turbidity of the bacterial suspension using sterile saline or MH broth to match the concentration of the standard turbidity tube. Complete the inoculation within 15 min to prepare a suspension at a 0.5 McFarland standard, which is equivalent to 1.5 × 10^ˆ^8 CFU/mL. Use a sterile cotton swab to dip into the above bacterial suspension. Press against the walls of the test tube a few times to remove excess liquid, then spread it evenly across the surface of the entire MH solid agar plate. Repeat this step twice more to ensure uniform distribution across the plate, and finally use the swab to cover the edges of the plate. Allow the inoculated plate to dry at room temperature for 3-5 min to ensure complete absorption of the bacterial suspension.

Using sterile tweezers, pick up the antibiotic susceptibility paper discs and place them on the surface of the plate, applying slight pressure with the tips of the tweezers to ensure they adhere flatly. Once placed, the position of the discs cannot be moved again. Incubate the plate with the discs in a constant temperature incubator at 35°C for 18–24 h. After incubation, measure the diameter of the inhibition zones using calipers.

#### Generating antimicrobial resistance resistomes and prevalence data

The method for generating Antimicrobial Resistance (AMR) molecular Resistomes, Variants, and Prevalence data involves utilizing the Resistance Gene Identifier (RGI), a specialized tool designed for detecting putative AMR genes from sequence data through models provided in the Comprehensive Antibiotic Resistance Database (CARD). This process includes analyzing molecular sequence data sourced from NCBI Genomes across 413 targeted pathogens, as well as genomic islands identified in Islandviewer. Each pathogen’s complete chromosome sequences, predicted genomic islands, plasmid sequences, and whole genome shotgun (WGS) assemblies are individually assessed by RGI to identify AMR genes.

Results are aggregated to calculate the prevalence of specific resistance genes, expressed as a percentage of occurrences across analyzed samples. Data is categorized using the Antibiotic Resistance Ontology (ARO) for clarity on the types of resistance mechanisms detected. The analysis distinguishes between "Perfect" matches, which correspond to curated reference sequences, and "Strict" paradigms that identify variants, ensuring reliable detection of functional AMR genes. The resultant Resistomes, Variants, and Prevalence data are updated regularly to reflect advancements in CARD curation and software, with applications ranging from bulk analyses of sequenced isolates to metagenomics studies and pathogen origin predictions.

### Quantification and statistical analysis

#### Data analysis and statistical testing

For Figures 4A and 5A, the *p*-values between groups were calculated using the Wilcoxon test through the R package ggpubr’s stat_compare_means function. The calculated *p*-values are displayed in the figures. For Figures 6A and 6B, significance was initially assessed using the Kruskal-Wallis test in GraphPad Prism v9.5 (GraphPad Software). Following a significant result, Dunn’s multiple comparisons test was applied to determine specific group differences. Significance levels are indicated by asterisks: ns denotes non-significance, ∗∗∗∗ indicates highly significant (*p* < 0.0001), ∗∗ indicates significant (*p* < 0.01), and ∗ indicates moderately significant (*p* < 0.05).

## Supplemental information

Document S1. Figure S1

Table S1. Genomic Sequencing Data for Acute, Chronic, and Healthy Control Phenotypes, related to Figure 1 This table provides a comprehensive dataset of sample information, including phenotype, sequencing statistics, mapping rates, and genome characteristics for various samples classified as acute or control

Table S2. Comprehensive Genomic Overview of Haemophilus influenzae Strains, related to Figure 1 This table compiles detailed information on various Haemophilus influenzae strains, their genomic features, and annotations from different sources, including NCBI and other sequencing projects

Table S3. Genetic Convergence Analysis of Acute and Chronic Sample Genes in NTHI, related to Table 1 This table lists genes that show convergence in expression or presence across different phenotypes, specifically highlighting those that are significant in acute versus chronic samples, along with their genomic annotations and intergenic regions

Table S4. KEGG Pathway Enrichment Analysis Results Summary, related to Figure 3 This table provides an overview of the enrichment analysis results, detailing various metabolic pathways and their associated statistical values such as *p* values and Corrected *p* values. It offers insights into the overrepresentation of specific pathways, indicating their significance in the context of the study

Table S5. Matching Table for Antibiotic Resistance Genes and Their Characteristics, related to Figure 5 This table presents a curated list of resistance genes matched with their corresponding antibiotics, resistance mechanisms, and gene families. It includes detailed definitions and information on how each gene confers resistance to specific drug classes, providing a valuable resource for understanding the genetic basis of antibiotic resistance

Table S6. Molecular Prevalence of Antimicrobial Resistance in Different Bacterial Species, related to Table 2 This table outlines the prevalence of antimicrobial resistance at the molecular level for a variety of species. It includes data on the presence of resistance genes in chromosomal DNA, plasmids, whole genome shotgun (WGS) sequences, and GenBank (GI) entries, offering insights into the genetic basis of antimicrobial resistance in these species

Table S7. Amino Acid Substitutions in PBP3 Associated with Antibiotic Resistance, related to Figure 6 This table examines the amino acid substitutions in the penicillin-binding protein 3 (PBP3), a key target for beta-lactam antibiotics. It presents the variations at specific positions (Asp-350, Ser-357, Ser-385, Leu-389, Val-562) for different sample IDs, categorizing them into Acute, Chronic, and Control groups to study the impact of these substitutions on antibiotic resistance
