# Supplementary material for: Comparative genomic study of non-typeable Haemophilus influenzae in children with pneumonia and healthy controls
Source: iScience. 2024 Nov 6;27(12):111330. doi: 10.1016/j.isci.2024.111330 (PMC11625288; doi:10.1016/j.isci.2024.111330)
Supplement: Document S1. Figure S1 [file mmc1.pdf]

iScience, Volume *27*

Supplemental information

Comparative genomic study of non-typeable *Haemophilus influenzae* in children with pneumonia and healthy controls

Deming Zhang, Wenjian Wang, Chunli Song, Tingting Huang, Hongyu Chen, Zihao Liu, Yiwen Zhou, and Heping Wang

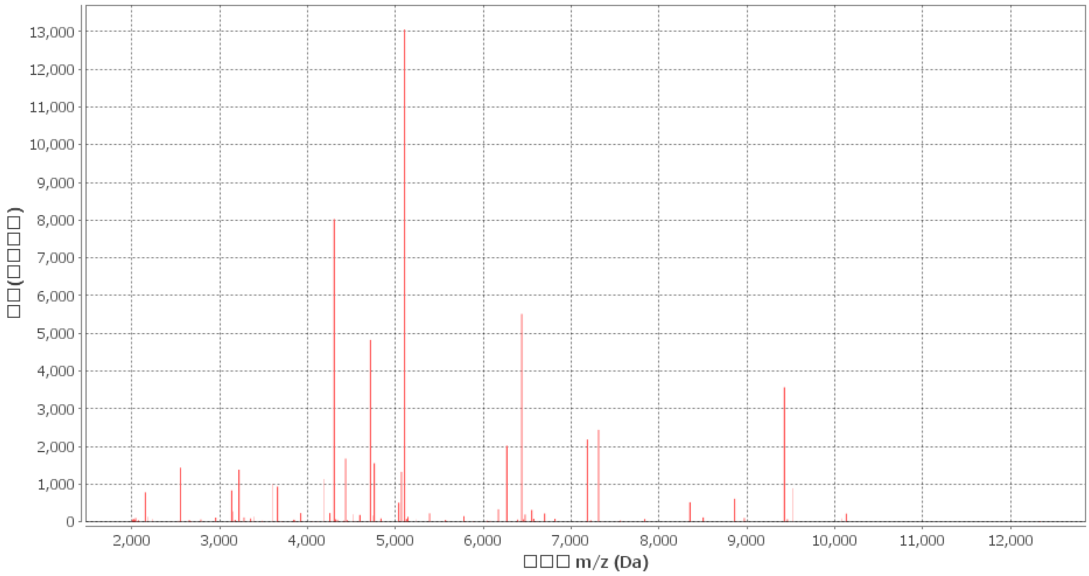

FiguerS1. The mass spectrum of Haemophilus influenzae. The x-axis denotes the mass-to-charge ratio (m/z), while the y-axis indicates the signal intensity (mV).
